# Measurement invariance across chronic conditions: a systematic review and an empirical investigation of the Health Education Impact Questionnaire (heiQ™)

**DOI:** 10.1186/1477-7525-12-56

**Published:** 2014-04-23

**Authors:** Michael Schuler, Gunda Musekamp, Jürgen Bengel, Sandra Nolte, Richard H Osborne, Hermann Faller

**Affiliations:** 1 Department of Medical Psychology and Psychotherapy, Medical Sociology and Rehabilitation Sciences Section, University of Würzburg, Klinikstr. 3, D-97070 Wuerzburg, Germany; 2 Department of Psychology, University of Freiburg, Engelbergerstraße 41, D-79085 Freiburg, Germany; 3 Medical Clinic, Department of Psychosomatic Medicine, Charité – Universitätsmedizin Berlin, Charitéplatz 1, D-10117 Berlin, Germany; 4 School of Health & Social Development, Deakin University, Burwood Campus, 221 Burwood Highway, Melbourne, VIC 3125, Australia

**Keywords:** Measurement invariance, Bias, Chronic disease, Generic questionnaire, Systematic review

## Abstract

**Background:**

To examine whether lack of measurement invariance (MI) influences mean comparisons among different disease groups, this paper provides (1) a systematic review of MI in generic constructs across chronic conditions and (2) an empirical analysis of MI in the Health Education Impact Questionnaire (heiQ™).

**Methods:**

(1) We searched for studies of MI among different chronic conditions in online databases. (2) Multigroup confirmatory factor analyses were used to study MI among five chronic conditions (orthopedic condition, rheumatism, asthma, COPD, cancer) in the heiQ™ with N = 1404 rehabilitation inpatients. Impact on latent and composite mean differences was examined.

**Results:**

(1) A total of 30 relevant studies suggested that about one in three items lacked MI. However, only four studies examined impact on latent mean differences. Scale means were only affected in one of these three studies. (2) Across the eight heiQ™ scales, seven scales had items with lack of MI in at least one disease group. However, in only two heiQ™ scales were some latent or composite mean differences affected.

**Conclusions:**

Lack of MI among disease groups is common and may have a relevant influence on mean comparisons when using generic instruments. Therefore, when comparing disease groups, tests of MI should be implemented. More studies of MI and according impact on mean differences in generic questionnaires are needed.

## Background

Generic questionnaires are based on the idea that important aspects of patients can be described across different chronic conditions. One such instrument, the Health Education Impact Questionnaire (heiQ™), aims to measure proximal outcomes of self-management programs across disease groups on eight disparate constructs, ranging from emotional distress to navigating the healthcare system. Ideally, the measurement properties of generic tools should be stable across disease-related characteristics, a property known as measurement invariance (MI) [1].

MI is often studied among gender, age or ethnic groups [2,3], but only little is known about MI across different chronic conditions. This paper helps to close this gap in the literature. The main research questions of this paper are, whether non-invariant items in generic questionnaires across different chronic conditions are a common finding and whether non-invariant items influence the validity of substantial statistical analyses with these questionnaires. First, the concept of MI and some important aspects of investigating MI are described. Second, a systematic review of studies that examined MI across different chronic conditions is presented. Third, the paper contains an empirical analysis of MI of the German version of the heiQ™. Results from the systematic review facilitate the interpretation of the results of the heiQ™ MI analyses.

### Measurement invariance

MI is the property of a measure being influenced systematically only by the construct that is intended to be measured. That is, no other characteristic of the persons being measured (for example gender or disease group) or the assessment context should have a systematic influence on the measurement results [4]. Therefore, persons with the same level in the construct of interest are expected to have the same numerical values in the measure. If MI does not hold between two or more groups in a measure, estimates of mean differences between these groups [5], correlations with other constructs [3] or selection decisions based on cut-off values [6] may be biased. It may even be questionable whether the instrument measures the same construct among comparison groups [5]. Therefore, MI is regarded as a prerequisite for group comparisons [1,7].

In the literature, a range of different concepts has been assigned to MI, for example “item bias” or “differential item functioning” (DIF) [4,7,8]. Although these concepts differ in some nuances from MI [4,5], they are used interchangeably for the purposes of this article. Furthermore, different statistical test procedures were developed to examine MI, some of which are based on observable variables, while others are based on latent variable models such as item response theory (IRT) or the common factor model [8,9]. Most of them follow the “…’matching principle’: systematic group differences in scores on a scale or item are considered as evidence of measurement bias only if group differences in scores remain among individuals who are all matched on the construct or latent variable being measured by the scale or item” ([9], p. S171). When using latent variable models, MI refers to invariant model parameters, e.g. factor loadings or item difficulties [7]. Unfortunately, different statistical methods can lead to different results; a “… true criterion …[to detect violations of MI did not]… stand up” ([10], p. S177). However, three aspects should be taken into account when studying MI: type of parameter [11], magnitude and impact [12].

*Type of parameter* refers to those parameters that can show DIF [8]. For example, multigroup confirmatory factor analysis (CFA) allows separating and testing different levels of MI, defined by the kind of model parameters that are restricted to be invariant across groups. To establish configural invariance, merely the number of latent variables and assignments of indicators on these latent variables have to be the same in all groups. Metric invariance is defined by invariant factor loadings, while scalar invariance is defined by metric invariance plus invariant intercepts. Finally, strict invariance is defined by additionally invariant residual (co-)variances [1,11,13]. If one or more parameters were non-invariant, partial invariance models can be tested, in which only some parameters on each level are restricted to be invariant [14]. At least (partial) scalar invariance has to be established to compare means of latent variables, while (at least partial) strict invariance is needed for mean comparisons in manifest variables to be permissible, e.g. composite scores [15-17]. Notably, in IRT-models, item discrimination parameters and item difficulty parameters can be viewed as counterparts of factor loadings and intercepts in common factor models, respectively [7,18]. DIF in item difficulty parameters is sometimes labeled “uniform” bias, while DIF in item discrimination parameters is called “non-uniform” bias [8]. DIF in residual variances is not tested in IRT models, as IRT models imply equal residual variances [8].

*Magnitude*, as defined here, refers to the size of differences in non-invariant parameters between groups, while *impact* designates the influence of non-invariant parameters on the main research questions, for example on mean differences in composite scores [10,19]. A researcher may detect a non-invariant factor loading of relevant magnitude (e.g., above 0.2 [20]) in one item of a scale. However, it is still possible that the mean group difference in the composite (scale) score is only marginally affected (small “impact”). The relationship between magnitude and impact is not quite clear. Some studies suggest that, in general, an increase in magnitude increases impact [3,5,21]; however, other aspects like the number of items in a scale, direction of invariant parameters, size of other model parameters or type of parameter may moderate this relationship. For example, Steinmetz [5] found that non-invariant intercepts may have a greater impact on mean comparisons compared to non-invariant factor loadings. Chen [3] showed that effects of multiple non-invariant parameters on mean differences may cancel each other out when the direction of invariant parameters is mixed, i.e. some parameter values are higher in the reference group and some are lower [10]. Although a general conclusion regarding the relationship between magnitude and impact is difficult to make, studies of measurement invariance should take both features into account.

In the last 20 years, many studies have been published to test MI in a variety of instruments in the social and health sciences. The majority of these studies examined MI in gender, age, language or culture [2]. Reviews of MI studies have shown that lack of MI is a common finding: In a review of cross-cultural MI, Chen [3] found that 74% of reviewed studies showed non-equal factor loadings in at least one item. According to Schmidt et al. [2] half of the reviewed studies tested partial invariance models, indicating that these studies found at least one non-invariant parameter.

In the health sciences, Teresi et al. [22] reviewed studies of MI for measures of depression, quality of life and general health. The main question was whether MI could be detected in the studied constructs (across any comparison groups) and whether the methods used to detect MI were appropriate. Only six of the reviewed studies examined MI across disease groups. Half of all studies did not examine all relevant types of MI. That is, magnitude and impact were often studied, but with differing results: Some studies reported only minor impact, while others reported non-ignorable impact. The review was restricted to methods based on observable variables and IRT models; methods based on the common factor model were not included.

To date, no systematic review examined whether disease group is associated with MI. However, MI across disease groups is of special interest in health science for several reasons: First, lack of MI might bias mean comparisons between different conditions in a generic construct. Second, lack of MI might also bias structural relationships between different constructs in different disease groups [3]. And finally, lack of MI might bias selection decisions based on cut-off values [6].

In the following section, a systematic review summarizes the knowledge in the scientific literature about MI in generic instruments across different chronic conditions. Then, an empirical investigation of MI among five different chronic conditions using the heiQ™ is presented. Afterwards, results of both studies are discussed.

## Systematic review

### Research questions

The systematic review tries to find out whether chronic condition should be regarded as a serious threat to MI in generic instruments. To explore this, the following main research questions were posed:

1) In general, how many items (in relation to the total number of items in an instrument) were regarded as non-invariant by the identified studies?

2) Do the identified non-invariant items have an impact on mean differences or other substantial statistical parameters?

Furthermore, the following questions should also be answered by the review:

How many studies can be identified that examined measurement invariance in generic instruments? Which constructs were examined, which chronic conditions were compared and which statistical methods used? What are the common explanations for lack of MI and what was recommend as the best ways to deal with it? Do some aspects of the studies (e.g. examined construct, number of comparison groups) correlate with the number of DIF-Items?

In contrast to other reviews [2,3,22,23], this review was not restricted to special statistical methods, for example CFA, or to a special time period.

### Methods

Studies were identified by searching electronic databases (Medline via both Pubmed and Ovid, PsycInfo) and by checking reference lists in identified studies and reviews [2,3,22,23]. Electronic search was performed on 29 August, 2012. As it was expected that results would contain many studies from areas other than health sciences (for example organizational research), results were filtered accordingly. Search and filter terms as well as inclusion and exclusion criteria are shown in Table 1.

**Table 1:** Search terms, filter terms and inclusion/exclusion criteria

Search terms	“Measurement invariance”, “factorial invariance”, “measurement equivalence”, “differential item functioning”, “item bias”
Filter terms	Chronic*, diagn*, patient*, rehab*, cancer, arthrit*, inflam*, diab*, rheum*, orthop*, respir*, asthm*, copd, health, quality of life, self management, self-management, empowerment, diseas*, depress*, anxiety, trauma, injury
Inclusion criteria	(a) empirical study of MI among different chronic conditions
	(b) generic questionnaire
	(c) adults
	(d) English or German language
Exclusion criteria	(a) only MI between factor correlations were studied, although scales were not combined to a total score;
	(b) instruments measure disease-related constructs such as disease-specific quality of life;
	(c) only specific subgroups of a chronic conditions were studied (e.g., patients with right- vs. left-hemispheric lesions).

Note: *was used as search term.

First, titles and abstracts were screened by one reviewer (MS). Then, full-text articles of all potentially relevant papers were retrieved. Two independent reviewers (MS; GM) determined eligibility of the studies.

Number of DIF-Items in relation to the whole number of items per questionnaire was determined (0-100%). Kendall’s τ correlation coefficients were computed between number of DIF-Items and examined construct, number of comparison groups, number of persons in the study, mean number of persons per comparison group.

### Results

#### Study selection

The search of electronic databases retrieved 4,017 references. After filtering, 2,014 studies remained and were evaluated on the basis of title and abstract. 91 potentially relevant references were identified. After examination of full-texts, a total of 30 studies were included. Interrater-reliability in the second step was moderate (Yules Y = 0.70) but all disagreements could be resolved by discussion. All relevant data of the studies are presented in Additional file 1: Table S1, online-supplement.

#### Constructs and instruments

A variety of constructs were examined by the reviewed studies: physical functioning [24-32], depression [33-36], illness-related distress [37], somatization [33], mental health [31], pain [38], manual ability [39], daily activities [40-42], mobility and self-care [43], quality of life [44], health status [45], breathless severity [46], kinesiophobia [47], dementia [48], patients opinion about their doctor [49], caregiver reactions [50], stigmatization [51], physicians empathy [52] and satisfaction [53].

Three instruments or scales (FIM, HAQ-DI, SF-36 Physical Functioning scale) were examined in more than one study. 23 of the examined measures were validated questionnaires or scales; six studies report the development of a questionnaire and two studies examined an item bank. One study examined two measures.

#### Number of patients and disease groups

In total, 34,608 patients were examined (M = 1,154, Md = 538). Most studies compared two (n = 13) or three (n = 11) disease groups, six studies compared five or more groups. The mean sample size per group was N = 343 (Md = 193). Generally, many different disorders were compared, while most studies included at least one neurological disorder.

#### Statistical methods

Most studies (n = 22) used methods based on IRT, six studies used common factor models and two studies used other statistical methods. Four studies investigated only metric or configural invariance. Only eight studies examined at least scalar invariance (i.e., both uniform and non-unifom DIF).

#### Number of invariant items, magnitude, impact and recommendations

On average, 31% (Md = 27%, Min = 0%, Max = 85%) of the items showed DIF. Excluding those studies that studied configural or metric MI only, DIF was found in 36% of the items. In 25 of the examined questionnaires (81%), at least one item showed DIF. 16 studies reported indicators of magnitude, e.g. item difficulty parameters in disease groups. However, 15 studies reported only p-values or no indicators of magnitude.

Of the 24 studies that identified at least one non-invariant item, only three examined impact on latent mean differences (none on composite mean differences). One of them reported statistically significant and relevant impact (d > 0.2, see below). However, 13 studies recommended adjusting for DIF or to be “cautious” when comparing means between or combining data across disease groups. Five studies examined correlations between adjusted and non-adjusted estimates. Generally, very high correlations (≥0.99) were reported indicating that structural relationships with other variables may not be affected when ignoring DIF. None of the studies examined impact on selection of patients according to cut-off-values.

#### Explanations for DIF

A total of 15 studies gave some explanations for non-invariant items. Most of them seemed to interpret DIF as reflections of real clinical differences. For example, in a study of Dallmeijer et al. [25], patients with stroke showed higher item difficulty in the SF-36 item ‘lifting/carrying groceries’ “… than patients with other multiple sclerosis or amytrophic lateral sclerosis, which is explained […] by the unilateral impairment of the arms of stroke patients” (p. 168). Besides, some authors also reported that undetected multidimensionality [27,36,37] or misworded items [27,41] might cause DIF and some further referred to other studies with similar results [28,32,34,43,45].

Studies examining physical functioning in a broader sense (e.g. including manual ability or daily activities) showed significant higher number of DIF-items (τ = 0.45). All other aspects of the studies showed no correlations with number of DIF-Items (all τ < |0.08|).

#### Summary

MI was examined across a variety of chronic conditions in many different constructs. DIF between disease groups in at least one item of a scale appears to be common. However, despite frequent recommendations to pay attention to items with DIF (or to delete them), only few studies explicitly examined impact of DIF on latent or composite mean differences.

## Empirical investigation of MI in the heiQ™

### Research question

The empirical investigation of MI in the heiQ™ was carried out among five chronic conditions (orthopedic conditions, rheumatism, asthma, COPD and cancer) and gender. Multigroup CFAs were used to test different levels of invariance. If non-invariant parameters were found, impact on latent and composite mean differences were examined via effect size measures.

### Methods

#### Sample

Patients from seven rehabilitation hospitals with a range of medical conditions (cancer, inflammatory bowel disease, orthopedic condition, respiratory disease, rheumatic disease) were included. All Patients completed heiQ™ at the beginning of inpatient rehabilitation. Parts of the patients were a subsample of patients from the study presented in [54]. The project was approved by the ethical review committee of Hannover Medical School (Nr. 5070). Participation in the study was voluntary and based on written informed consent.

## The Health Education Impact Questionnaire (heiQ™)

The heiQ™ was developed in Australia and measures proximal outcomes of self-management programs. It contains 40 items (4-point response scale) across eight independent scales: *Positive and active engagement in life*, *Health directed activities*, *Skill and technique acquisition*, *Constructive attitudes and approaches*, *Self-monitoring and insight*, *Health service navigation*, *Social integration and support*, and *Emotional distress.* The scales were developed using CFA and item response theory [55]. In the German version, the factorial structure was replicated with only minor adjustments (i.e. freeing error covariances between two items in five scales each) [54]. Generally, higher values in the heiQ™ scales indicate better status, except for *Emotional distress*, in which higher values indicate higher distress. The scales show appropriate associations with constructs like subjective health, depression or cognitive and emotional representations of an illness [54]. The heiQ™ can be used to display the effects of self-management programs in outpatient and community settings [56-59] and was recently used to guide a Cochrane Review of self-management programs [60]. Further information on the heiQ™ can be found in [55,61].

Both in Australia and in Germany, factorial validity was examined in about 1200 rehabilitation patients with a variety of chronic conditions, respectively. Nolte et al. [62] examined MI over time (response-shift [63]) in the heiQ™. Although using a sample that included different chronic conditions, this study suggested remarkably stable psychometric properties of the heiQ™ over time. However, statistical models can show good fit values in heterogeneous samples even though subsamples may have different parameter values [64]. Therefore, the results of these studies cannot be interpreted as evidence of MI between chronic conditions.

### Data analysis

To test different levels of MI, several multigroup CFAs were computed. All analyses were done with Mplus Version 6.1 [65] using robust maximum likelihood estimator. MI was examined for each scale separately. The measurement models of the German heiQ™ were used as baseline models to test configural invariance. To identify the models, the procedure suggested by Yoon & Millsap [66] was used: For testing configural invariance, the factor loadings of one indicator item was set to 1 (the same item in all groups) and the mean of the latent variable was fixed to zero in all groups. All other parameters were free to vary among groups. To test for metric invariance, the variance of the latent variable in the reference group was set to 1 and all factor loadings were fixed to be invariant between groups (the mean of the latent variable was still fixed to zero in all groups). Scalar invariance was tested by additionally restricting all intercepts to be equal between groups; the mean of the latent variable was still fixed to zero in the reference group but was allowed to vary across all other groups. Finally, strict invariance was tested by restricting all residual variances (and covariances between residual terms) to be invariant among all comparison groups.

Configural invariance was assessed by global evaluation of model accuracy using chi^2^-test as well as the model fit indices Comparative fit index (CFI) and Root mean square error of approximation (RMSEA). For model fit to be interpreted as at least ‘acceptable’, CFI should be close to 0.95 or above and RMSEA close to 0.06 or below [67]. Following Saris et al. [20], metric, scalar and strict invariance of parameters (factor loadings, intercepts, residual variances) were evaluated by expected parameter changes (EPC) and modification indices using the software JruleMplus [68]. A modification index can be regarded as a test statistic for a significance test (with 1 degree of freedom) for a misspecification (e.g., a fixed factor loading) and an EPC offers an estimate of that misspecification. Using the formulas provided by Saris et al. [20], we tested whether a potential misspecification exceeds a reference value δ. δ is determined by the researcher and represents the size of a misspecification regarded as relevant. In studies of MI, δ represents the minimal difference in factor loadings, intercepts etc. among comparison groups that are regarded as meaningful, respectively. In other words, δs represent the lower limits of magnitudes of non-invariant parameters while EPCs are estimates of actual magnitudes. However, there are no rules of thumb for choosing appropriate critical values for equally constraints [69,70]. For example, Steinmetz [5] found that in scales with four or six items, differences in (unstandardized) factor loadings of 0.3 in one or two items may have only small, but differences in intercepts of 0.075 times the scale range may have considerable impact on latent and composite mean differences. To be on the safe side, δ was fixed on δ =0.15 for (unstandardized) factor loadings and error variances and to be 0.04 times the scale range of the latent variable (δ = 0.12) for intercepts. Furthermore, the conclusion drawn by the analysis must take the power of the modification index test into account, which can be computed for every combination of modification index, EPC, δ and significance level alpha (which was fixed at alpha = 0.05 in this study). We followed Saris et al. [20] and regarded results based on tests with low power (<0.8) and nonsignificant modification indices (i.e. modification indices < 3.84), as “inconclusive”, which means that it is not possible to decide whether the misspecification exceeds δ or not, i.e. whether the examined parameter is invariant or not. For these parameters, impact on mean differences was not examined (see below). For more details on the outlined procedure, see [20,69,71]. Whenever DIF was found in a parameter, the parameter was set free and partial invariance models were tested. When more than one parameter was found to be non-invariant, the parameter with the highest EPC was set free and the new model was tested. When JruleMplus still identified non-invariant parameters, the procedure was repeated until no further misspecification was indicated.

The impact of non-invariant parameters on latent mean differences was tested via comparison of mean group differences between partial measurement invariance models (PIM) and strict invariance model (SIM). PIM were regarded as the “true” models, while SIM (wrongly) assumes that all parameters were invariant across all groups. Standardized mean differences in latent variables [72] between comparison groups were computed in both SIM (SI_Diff_) and PIM (PI_Diff_). Then the term ES_SI-PI_ = SI_Diff_-PI_Diff_ was computed. ES_SI-PI_ represents the size of misestimating the standardized mean difference between two comparison groups if a SIM is chosen. Because SI_Diff_ and PI_Diff_ are comparable to Cohen’s d [72], ES_SI-PII_ is also a standardized value. Following Cohen [73], values for ES_SI-PI_ above |0.2| are regarded as a relevant impact of non-invariant parameters on latent mean differences.

To study the impact on group differences in composite means, we first computed standardized effect sizes (Cohen’s d) between comparison groups in composite scales in two ways: One (ALL_Diff_) by using all items of a scale (and thus implicitly assuming strict MI), and one by using a reduced scale with only strictly invariant items between two comparison groups (RED_Diff_). Then the terms ES_PI-ALL_ = PI_Diff_-ALL_Diff_ and ES_PI-RED_ = PI_Diff_-RED_Diff_ were computed. Assuming that PI_Diff_ represents the “true” difference between comparison groups, ES_PI-ALL_ and ES_PI-RED_ indicate misestimation of group differences by using ALL_Diff_ or RED_Diff_. Again, values for ES_PI-ALL_ and ES_PI-RED_ above |0.2| are regarded as relevant. Furthermore, by comparing ES_PI-ALL_ and ES_PI-RED_, it was examined whether deleting non-invariant items led to an improved estimation of group differences.

### Results

#### Sample

The sample comprised N = 1404 German rehabilitation patients (42% women, mean age = 56.4 years (SD = 12.2)) with different chronic conditions. All patients with orthopedic conditions (e.g. chronic back pain) (n = 180), rheumatism (e.g. psoriatic arthritis, ankylosing spondylitis) (n = 312), asthma (n = 225) and COPD (n = 118) as well as n = 136 cancer patients were from the study presented in [54]. The sample was supplemented by an additional n = 433 cancer patients who also filled out the German heiQ™ at the beginning of their inpatient rehabilitation. From all cancer patients, n = 215 were diagnosed with prostate cancer, n = 217 with colon or rectum cancer and n = 137 had another type of cancer. When analyzing MI across gender, patients with prostate cancer were excluded.

#### Number, kind and magnitude of non-invariant parameters

##### Gender

In two scales, one item each did not show scalar invariance: Item 10 in *Positive and active engagement in life* (EPC = 0.12) and Item 9 in *Health directed activities* (EPC = 0.16). All other scales showed strict invariance across gender.

##### Disease groups

Table 2 shows fit indices for strict and partial invariance models and Table 3 shows results of invariance tests of specific parameters. One heiQ™ scale proved to be strictly invariant between all five disease groups (*Social integration and support*). Three scales (*Emotional distress*, *Skill and technique acquisition, Health directed activities*) showed at least scalar invariance among four conditions. *Health service navigation* was strictly invariant between patients with orthopedic conditions and rheumatism on the one hand and patients with asthma, COPD, and cancer on the other. *Constructive attitudes and approaches* showed strict invariance in three conditions (cancer, asthma, and orthopedic conditions). *Active engagement in life* showed only metric invariance between all conditions, but at least scalar invariance among rheumatism, cancer, and COPD. *Self-monitoring and insight* showed metric invariance among patients with orthopedic conditions and cancer on the one hand and patients with asthma, COPD, and rheumatism on the other hand. Scalar invariance could not be established across any chronic condition group in this scale; however, a partial invariance model could be established. A total of 14 items (35%) showed DIF in any analyzed parameter level in at least one disease group. However, 2–3 items showed DIF only in residual variances, which do not affect mean differences between groups. Point estimates of EPCs for factor loadings and residual variances were only slightly above the defined values for δ; EPCs for intercepts ranged between 0.10 and 0.34.

**Table 2:** Fit-values for strict invariance models (SI) and partial invariance models (PI) among chronic conditions

**Scale**	**Model**	**Chi**^**2**^**(df)**	**p**	**CFI**	**RMSEA**
**Positive and active engagement in life**	SI	212.21 (99)	<0.001	0.868	0.079
	PI	121.99 (68)	<0.001	0.947	0.053
**Health directed activites**	SI	85.72 (49)	<0.001	0.975	0.052
	PI	69.16 (47)	0.019	0.985	0.041
**Skill and technique acquisition**	SI	62.966 (50)	0.103	0.986	0.030
	PI	45.08 (47)	0.552	1.000	0.000
**Constructive attitudes and approaches**	SI	164.04 (88)	<0.001	0.940	0.063
	PI	142.26 (72)	<0.001	0.952	0.059
**Self-monitoring and insight**	SI	434.91 (108)	<0.001	0.696	0.104
	PI	141.97 (92)	<0.001	0.953	0.044
**Health service Navigation**	SI	259.45 (76)	<0.001	0.870	0.095
	PI	160.20 (72)	<0.001	0.941	0.066
**Social integration and support**	SI	155.52 (76)	<0.001	0.960	0.061
	PI	--	--	--	--
**Emotional distress**	SI	255.75 (108)	<0.001	0.944	0.070
	PI	208.14 (105)	<0.001	0.961	0.051

Notes: SI: Strict invariance model; PI: Partial invariance model (non-invariant parameters see Table 4).

**Table 3:** Results of tests of MI across five chronic conditions, arranged by type of parameter

**Scale**	**Configural MI**	**Metric MI**			**Scalar MI**			**Strict MI**		
		**Diag**	**Item**	**EPC**	**Diag**	**Item**	**EPC**	**Diag**	**Item**	**EPC**
**Positive and active engagement in life**	✓		✓		ortho	2	-0.24	copd	2	0.13
					ortho	5	-0.18			
					asthma	2	-0.17			
					asthma	10	0.12			
**Health-directed activities**	✓		✓		copd	19	0.24		(✓)	
**Skill and technique acquisition**	✓		✓		copd	23^a^	-0.17	ortho	30	0.19
					asthma	23^a^	-0.09			
**Constructive attitudes and approaches**	✓	copd	36	0.16	rheuma	36	0.13		(✓)	
**Self-monitoring and insight**	✓	ortho	11^a^	-0.14	ortho	3	-0.10	ortho^b^	11	-0.13
					ortho	17	-0.13			
					asthma	3^a^	-0.22			
					asthma	17^a^	-0.20			
					copd	3^a^	-0.31			
		cancer	11^a^	-0.15	copd	6	0.34			
					copd	17^a^	-0.21			
					cancer	6	-0.28			
**Health-service navigation**	✓		✓		ortho	33^a^	-0.21		(✓)	
					rheuma	33^a^	-0.29			
**Social integration and support**	✓		✓			✓			✓	
**Emotional distress**	✓		✓		cancer	12	-0.19	ortho	7	-0.16

Notes: MI: measurement invariance; numbers (“Item”) represent non-invariant heiQ™ items in the mentioned disease group (“Diag”), followed by EPC (Expected Parameter Change) with ortho = orthopedic conditions, rheuma = rheumatism; ✓: all parameter invariant; (✓): no new DIF parameter, but parameters of items with DIF in a former stage were set free; ^a^invariant parameter in subgroups (for example item 3 has the same intercept in COPD and asthma); ^b^in item 11, orthopedic group and cancer group show same factor loadings and intercept, but differ in residual variances.

Because of limited power, for some parameters in each scale it could not be concluded whether they exceed δ or not. However, point estimates of EPCs for these parameters were mostly low (a table with all EPCs and modification indices as well as power estimates may be offered on request).

#### Impact on latent mean differences

##### Gender

In both scales showing one non-invariant item each, no relevant impact on latent or composite mean differences was found (*Positive and active engagement in life*: ES_SI-PI_ = 0.08, ES_PI-ALL_ = 0.13, ES_PI-RED_ = 0.06; *Health directed behavior*: ES_SI-PI_ = 0.06, ES_PI-ALL_ = 0.09, ES_PI-RED_ < 0.01).

##### Disease groups

Table 4 shows coefficients for the impact of non-invariant items on both latent and composite mean differences among all five conditions for the two scales *Positive and active engagement in life* and *Self-monitoring and insight*. In all other scales, no relevant impact was found (exact values are shown in Additional file 2: Table S2, online-supplement).

**Table 4:** **True standardized mean differences (PI**_**DIFF**_**) and impact of non-invariant items on latent (ES**_**SI-PI**_**; lower triangle) and composite (ES**_**PI-ALL**_**, ES**_**PI-RES**_**, upper triangle) mean differences**

**Disease group**		**Ortho**	**Rheu**	**Asthma**	**COPD**	**Cancer**	
**Positive and active engagement in life**							
**Ortho**			0.32^a^	0.27^a^	0.31^a^	0.24^a^	**ES**_**PI-ALL**_
			0.09	0.17	0.06	0.03	**ES**_**PI-RED**_
**Rheu**	**PI**_**Diff**_	0.59		0.08	0.02	0.10	**ES**_**PI-ALL**_
	**ES**_**SI-PI**_	0.27^a^		0.14	0.05	^b^	**ES**_**PI-RED**_
**Asthma**	**PI**_**Diff**_	0.20	-0.43		0.06	0.02	**ES**_**PI-ALL**_
	**ES**_**SI-PI**_	0.28^a^	-0.02		0.09	0.04	**ES**_**PI-RED**_
**COPD**	**PI**_**Diff**_	0.47	-0.13	0.27		0.08	**ES**_**PI-ALL**_
	**ES**_**SI-PI**_	0.27^a^	-0.13	0.01		0.12	**ES**_**PI-RED**_
**Cancer**	**PI**_**Diff**_	-0.03	-0.64	-0.24	-0.52		
	**ES**_**SI-PI**_	0.28^a^	> - 0.01	0.01	> - 0.01		
**Self-monitoring and insight**							
**Ortho**			0.11	0.22^a^	0.12	0.10	**ES**_**PI-ALL**_
			0.02	0.06	0.02	0.04	**ES**_**PI-RED**_
**Rheu**	**PI**_**Diff**_	0.24		0.32^a^	0.22^a^	0.21^a^	**ES**_**PI-ALL**_
	**ES**_**SI-PI**_	0.08		0.13	0.08	0.10	**ES**_**PI-RED**_
**Asthma**	**PI**_**Diff**_	-0.34	-0.56		0.09	0.13	**ES**_**PI-ALL**_
	**ES**_**SI-PI**_	-0.05	-0.13		0.03	0.02	**ES**_**PI-RED**_
**COPD**	**PI**_**Diff**_	-0.16	-0.38	0.16		0.03	**ES**_**PI-ALL**_
	**ES**_**SI-PI**_	-0.03	-0.10	0.03		0.02	**ES**_**PI-RED**_
**Cancer**	**PI**_**Diff**_	-0.31	-0.55	0.04	-0.15		
	**ES**_**SI-PI**_	<0.01	-0.08	0.06	0.02		

Notes: Ortho: orthopedic condition; Rheu: rheumatism; PI_Diff_: Estimations of latent mean differences in partial invariance models; ES_SI-PI_: Difference in latent mean differences between strict and partial invariance models; ES_PI-ALL_: Difference between latent mean differences in partial invariance models and composite mean differences using all items of a scale; ES_PI-RED_: Difference between latent mean differences in partial invariance models and composite mean differences using only items with pairwise non-invariant parameters; ^a^relevant misestimation (ES > |0.2|); ^b^no item with DIF between groups.

In *Positive and active engagement in life*, all comparisons among orthopedic patients and other disease groups in latent means were affected in a relevant manner by non-invariant parameters (all ES_SI-PI_ > 0.26). Accordingly, using the composite scale with all items, differences were also clearly misestimated (0.24 ≤ ES_PI-ALL_ ≤ 0.32). Deleting the non-invariant items in the composite scale reduces this bias (0.03 ≤ ES_PI-RED_ ≤ 0.17). Ignoring non-invariant parameters did not have a relevant influence on any other latent or composite comparisons in this scale (all ES_SI-PI_ and ES_PI-ALL_ < |0.2|).

Despite showing a complex pattern of non-invariant parameters, ignoring them in *Self-monitoring and insight* did not lead to relevant misestimation of latent mean differences (0.01 ≤ ES_SI-PI_ ≤ 0.13). However, using composite scales with all items of the scale led to a relevant misestimation of mean differences in four comparisons (orthopedic vs. asthma, rheumatism vs. asthma, rheumatism vs. COPD, rheumatism vs. cancer). Again, deleting non-invariant items in the composite scales reduces this bias (all ES_PI-RED_ < |0.13|).

## Discussion

As far as we know, this is the first review of studies on MI in generic constructs across disease groups and the first review on MI not restricted to a specific statistical technique. Studies of MI among diagnostic groups have become more prevalent in the last years; only one of the reviewed studies was published before 2000. Disease group appears to be increasingly recognized as an important factor that may influence MI in a variety of generic constructs.

At first glance, the results of both the review and the analyses of the heiQ™ seem to confirm the assumption that MI is an important aspect when applying generic instruments across disease groups. Over 80% of the examined questionnaires showed at least one item with non-invariant parameters; the mean proportion of non-invariant items was 36% (excluding studies that examined configural or factorial invariance only). Presumably, the actual number of distortions in MI may even be higher. First, only a few studies examined both uniform and non-uniform bias. Second, apart from the studies in the review, many studies did not examine MI directly, but analyzed factor structure and other parameters of a measure in specific conditions and compared results descriptively with results of other studies. These studies may underestimate lack of MI; hence, the number of items showing DIF may even be higher. Likewise, 35% of the heiQ™ items showed DIF in at least one disease group.

However, items showing DIF did not always have an impact on the main research questions. It is difficult to assess whether non-invariant items of the reviewed studies had relevant impact as only three studies [25,26,30] examined influences on (latent) mean differences, with only one showing a relevant impact [25]. Five studies examined impact of items with DIF on structural parameters indirectly, i.e. impact was explored via correlations of DIF-adjusted and non-adjusted values. Finally, none of the studies examined impact on either composite mean differences or on accuracy of selection. In contrast, we carried out a more detailed analysis of the heiQ™ where we demonstrated that seven scales included items with DIF. However, only few parameters were non-invariant in five of these scales and none of them had a relevant influence on latent or composite mean comparisons.

The remaining two heiQ™ scales, however, showed several non-invariant parameters among disease groups. Indeed, partial invariance models among disorders could be proven but at least some group comparisons were affected by non-invariant parameters.

*Self-monitoring and insight:* A complex pattern of non-invariant factor loadings and intercepts among the five disease groups indicating partial invariance was found in this scale. This pattern may best be interpreted as a reflection of clinical differences among disease groups. For example, item 11 asks patients whether they know how and when to take their medicine. However, use of medication may have greater importance to patients in some conditions (e.g. rheumatism or asthma) than in others (e.g. chronic back pain). Another example is item 3 asking patients about their self-monitoring activities. Asthma patients show a lower intercept (difficulty) than both rheumatic and cancer patients in this item. Asthma patients may well be more motivated to monitor their health than rheumatic patients or cancer patients are, because an immediate intervention (e.g. using an inhaler) has a direct effect on their health status. Interestingly, despite the complex pattern of non-invariant items, only a small impact on latent means was detected. Still, some composite mean comparisons were clearly affected.

*Active engagement in life:* Patients with orthopedic conditions (i.e. chronic back pain) showed lower intercepts in item 5 (“I try to make the most of my life”) and item 2 (“Most days I’m doing some of the things I really enjoy”), resulting in a relevant impact on latent and composite mean differences. A possible explanation may be that psychosocial factors play a larger role in chronic back pain than in other conditions; therefore, patients may pay more attention to stress-reducing activities. However, this explanation is highly speculative. More research is needed to clarify these issues.

The review showed that a higher amount of non-invariant items was found in studies that examined physical functioning. A possible explanation might be that people with different somatic diagnoses differ in how strong different areas of activity are affected. A general hypothesis would be that the more a measured construct is influenceable by the kind of disease, the higher is the probability that indicators of the construct show DIF between disease groups. The high number of items showing DIF in *Self-monitoring and insight* would be in line with this hypothesis.

The results also clarified that DIF should not only be regarded as an aspect of an item as such, but, in many cases, as an interaction between item and disease group. Many heiQ™ items showed DIF only in one of the five comparison groups. Similar results were presented in some reviewed studies. For example, many items in one study [43] showed DIF only between two out of three compared disease groups.

### Limitations

Many statistical methods have been developed to examine MI, but it remains unclear which method is the most appropriate one to use. For example, the statistical method used in the present study differs from the often recommended CFA-procedure that tests for MI by comparing global fit-values (for example chi^2^-difference test or differences in CFI) [4,11,13,74]. The outlined procedure in this study may be more sensitive to detect “truly” non-invariant items, because the magnitude of the EPC and the power of modification indices are taken into account. However, values of EPC and MI depend on the correctness of all other model parameters [20]. If more than one parameter is non-invariant, EPCs and MIs may also be misleading. Furthermore, the power for each examined parameter varied greatly, due to different sample sizes in disease groups or different sizes of model parameters in different heiQ™ scales. This may have influenced the presented results. More studies that compare different procedures for examining invariance are needed.

As (non-)invariance is a continuum rather than a dichotomous state [10], the results of all studies about MI highly depend on the choice of adequate cut-off-values for magnitude and impact, respectively. We used very strict cut-off values in the present study, leading to a high sensitivity to detect potential non-invariant items. Choosing other cut-of-values may have reduced or increased the number of DIF-items. Higher cut-off values may also reduce the numbers of inconclusive comparisons. Up to now, only little guidance can be found in the literature for selecting values for δ. Furthermore, few studies proposed effect size measures for estimating impact [75,76]. More empirical and simulation studies are needed to help researchers define relevant cut-off values for both magnitude and impact for all statistical approaches examining MI (for another solution to these problems using Bayes analyses, see [77]).

Furthermore, it is not known whether results of MI-analyses between disease groups are consistent across languages and cultural groups. Future work that simultaneously explores cross-cultural and disease-specific MI issues seems warranted to generate information on the presence and magnitude of bias in evaluating chronic disease programs across countries.

## Conclusion

Since most heiQ™ scales showed strict invariance across gender and non-invariant items did not affect mean difference between men and women in a relevant manner, the heiQ™ can be used to compare men and women without any adjustments. In six scales, comparisons of mean differences among disease groups were also not affected by invariant items, again suggesting that no adjustments have to be made. This study showed that the heiQ™ is a robust tool for studies within disease groups and is likely to be an unbiased measure in controlled studies with balanced samples across disease groups. However, in studies with unbalanced disease groups the *Self-management and insight* and *Positive and active engagement in life* scales should be checked for distortions of MI. To adjust for MI, we suggest comparing latent means of partial invariance models instead of deleting non-invariant items [5].

This study demonstrates that a lack of MI across disease groups in generic instruments is common; maybe more common than in other socio-demographic variables like gender. However, its clinical impact remains unclear. Generally, routine examinations of the presence of invariance seems to be warranted, particularly when testing hypotheses around disease group differences and in settings where researchers are seeking to develop generic instruments for applications across disease groups [10]. This field will be advanced by more systematic studies of MI across disease groups and other clinically relevant variables. This entails simulation studies focusing particularly on the relationship between magnitude and clinical impact of DIF as well as qualitative methods to elucidate sources of DIF.

## Competing interests

The authors declare that they have no competing interests.

## Authors’ contributions

MS is the principal investigator, developed the study, performed the statistical analysis and is the main author of the manuscript. MS and GM performed the systematic review. MS, GM and HF drafted the manuscript. JB, SN and RO contributed to the design of the study and helped drafting the manuscript. All authors revised and approved the final manuscript.

## Author’s information

Michael Schuler: http://www.psychotherapie.uni-wuerzburg.de.

## Supplementary Material

Additional file 1: Table S1 Results of the systematic review. Click here for file

Additional file 2: Table S2 True mean differences (PIDIFF) and impact of non-invariant items on latent (ESSI-PI; white) and composite (ESPI-ALL, ESPI-RES, grey) mean differences. Click here for file
