# Supplementary material for: Measurement invariance across chronic conditions: a systematic review and an empirical investigation of the Health Education Impact Questionnaire (heiQ™)
Source: Health Qual Life Outcomes. 2014 Apr 23;12:56. doi: 10.1186/1477-7525-12-56 (PMC4021509; doi:10.1186/1477-7525-12-56)
Supplement: Additional file 1: Table S1 — Results of the systematic review. [file 1477-7525-12-56-S1.pdf]

Table A1: Results of the systematic review

Article	Measure (abbr.)	Construct	Method	Typeof DIF	Disease group[1]: N	Number of DIF-Items (%)	Magnitude	Impact on mean differences	Recom-mendation	Status[2]
Atkinson et al., 2011	BPI	Pain	CFA	metric	HIV: 209<br>Cancer: 155<br>Total: 364	0 (0%)	no	n.n.	n.n.	vq
Bode et al., 2003	SF-36 PF	Physical functioning	IRT (RRS)	uniform	Cancer: 399<br>HIV: 170<br>Stroke: 328<br>Total: 897	5 of 10 (50%)	no	no	delete DIF-Items	vs
C. C. Chen & Bode 2010	MAM-36	Manual Ability	IRT (Rasch)<br>multiple t-test	uniform	Neuro: 129<br>Musco: 206<br>Total: 335	14 of 36 (39%)	yes	no	no adjustments	vq
Chien et al., 2009	PFQ	Patients opinions about their doctor	IRT (RRS)<br>d >0.5 logit	uniform	Internal: 109<br>Surgical: 97<br>Obst & Gyn.: 41<br>Pediatric: 23<br>Others: 129<br>Total: 399	9 of 23 (39%)	yes	no	delete DIF-Items	vq
Coster et al., 2004	Personal Care and Instrumental Activities Item Set	Daily activities	IRT (PCM)<br>multiple t-tests	uniform	Neuro: 159<br>Musco: 136<br>Med comp: 182<br>Total: 477	8 of 62 (13%)	yes	no	no adjustments	ib
Dallmeijer et al., 2005	SF-36 PF	Physical functioning	IRT (PCM)<br>Residual Anova	uniform<br>non-uniform	Stroke: 198<br>MS: 151<br>ALS: 194<br>Total: 543	7 of 10 (70%)	yes	relevant	adjustment with stroke;<br>no adjutment MS vs. ALS	vs
Dallmeijer et al., 2007	FIM	Physical functioning	IRT (RRS)<br>multiple t-tests	uniform	Stroke: 295<br>MS: 150<br>TBI: 88<br>Total: 533	13 of 18 (72%)	yes	yes:<br>small	adjustment	vs

Farin & Fleitz, 2009	MOSES-Physician	Mobility<br>Self-care	IRT (PCM)<br>multiple t-test	uniform	Musco: 549<br>Neuro: 258<br>Cardio: 212<br>Total: 1119	28 of 47 (60%)	no	no	not combining	dq
Given et al., 1992	CRA	Caregiver Reactions	CFA	metric;<br>factor covariances	Alzheimer: 101<br>Cancer: 276<br>Total: 377	0 (0%)	n.n.	n.n.	n.n.	dq
Haley et al., 2004	AM-PAC	Daily activities	IRT (RRS)<br>multiple t-tests	uniform	Neuro: 155<br>Musc: 138<br>Med comp: 184<br>Total: 476	7 of 41 (17%)	yes	no	rewording;not deleting	dq
Hart, Mioduski & Stratford, 2005	SCL-90-R: Items for depression and somatization	Depression<br>Somatization	IRT (GRM)<br>ordinal regression	uniform<br>non-uniform	Ortho: 8834<br>Neuro: 328<br>Med: 186<br>Indust: 895<br>CPM: 655<br>Total: 10,898	1-2 of 17<br>[6-11%]	no	no	no adjustments	vs
Lindeboom et al., 2004	SIP	Physical functioning	IRT (extended Rasch)	uniform	Stroke: 653<br>Internal: 854	58 of 127 (46%)	yes	no	none	dq
Lundgren-Nilsson et al., 2006	FIM – motor scale	Physical functioning	IRT (PCM)<br>Residual Anova	uniform<br>non-uniform	Stroke: 157<br>SCI: 157<br>TBI: 157<br>Total: 471	including spinal: 11 of 13 (85%); without spinal: 6 of 13 (46%)	no	no	adjustment	vs
Moorer et al., 2001	RAND-36	health status	IRT (Mokken)	uniform	COPD: 148<br>MS:352<br>Rheuma: 223<br>Total: 723	4 of 36 (11%)	no	no	no adjustments	vq
Pickard et al., 2006	CES-D	Depression	IRT (RRS)<br>multiple t-tests	uniform	Dep & Stroke: 32<br>Dep: 366<br>Total: 398	4 of 20 (20%)	yes	no	no adjustments	vq
Prieto, Delgado, Perea & Ladera, 2011	MMSE	Dementia	IRT (Rasch)<br>multiple t-tests	uniform	PD: 119<br>Alzheimer: 110<br>Total: 321	6 of 30 (20%)	no	no	not comparing	vq

Rao et al., 2009	SSCI	Stigmatization	Mantel-Haenszel & Liu-Agresti cumulative common log odds ratio	uniform	Epilepsy: 165<br>Stroke: 190<br>Total: 355	4 of 24 (17%)	yes	no	no adjustments	dq
Reilly, Bowden, Bardenhagen & Cook, 2006	BDI	Depression	CFA	configural<br>metric<br>skalar<br>strict	Epilepsy: 187<br>other Neuro: 150<br>Total: 337	0 (0%)	no	n.n.	n.n.	vq
Roelofs et al., 2007	TSK	Kinesiophobia	CFA	configural (factor loadings and residuals fixed)	Total: 2825 [allocation to comparison groups not fully clear]	0 (0%)	no	no	no adjustments	vq
Steultjens et al., 2012	WOMAC-PF	physical functioning	IRT (RRS)<br>ordinal regression	uniform<br>non-uniform	OA: 288<br>PD: 191<br>LSP: 169<br>Total: 648	4 of 17 (24%)	yes	no	no adjustments	vs
Taylor & McPherson 2007	SF-36 PF<br>HAQ-DI	Physical functioning	IRT (RRS)<br>multiple t-tests	uniform	RA: 142<br>PA: 134<br>Total: N=276	SF-36: 4 of 10 (40%)<br>HAQDI: 3 of 8 (38%)	yes	no	SF-36 PF preferable	vs
van Groen et al., 2010	HAQ-DI	Physical functioning	IRT (gPCM)<br>LM statistic	uniform	RA: 691<br>OA: 125<br>Gout: 102<br>Total: 818	4 of 8 (50%)	yes	yes:<br>small<br>not sign.	adjustements	vs
Waller et al., 2005	BDI-II	Depression	Logistic regression	uniform<br>non-uniform	Breast Cancer: 267<br>Dep: 294<br>Total (woman): 561	15 of 21 (71%)	NCDIF	no	caution when interpreting breast cancer patients	vq
Wann-Hansson, et al. 2008	NHPD	illness-related distress	IRT (Rasch)<br>Residual anova	uniform<br>non-uniform?	PD: 258<br>PAD: 215<br>Total: 473	7 of 24 (29%)	no	no	no adjustments	vs

Weisscher, Glas, Vermeulen & De Haan, 2010	ALDS	daily activities	IRT (2-PL)) LM statistic	uniform	Neuro: 497 Internal: 786 Total: 1283	18 of 77 (23%)	yes	yes; small sign.	adjustment	ib
Wirtz, Boecker, Forkmann & Neumann, 2011	CARE	Physicians empathy	IRT (RRS) multiple t-Tests	uniform	Cancer - Bronchial: 28 Colorectal: 18 Prostate: 54 Oesophagal: 35 Breast: 109 Skin: 68 Other:7 Total: 309	5 of 10 (50%)	yes	no	adjustment	vq
Wong, Nordstokka, Gregorisch & Perez-Stable, 2009	ChPSQ-9	Satisfaction	CFA	metric; factor covariances; residual variances	Breast Cancer: 249 Lung Cancer: 334 Total: 583	1 of 9 (11%)	no	no	no adjustments	vq
Yao & Wu, 2005	WHOQOL-BREF	Quality of Life	CFA	metric	Pulmonary: 235 Hypertension: 487 Peptic ulcer: 487 Sinusitis: 376 Liver disease: 500 Total: 2726	0 (0%)	no	n.n.	n.n.	vq
Yorke, Horton, & Jones, 2012	Dyspnoea-12	breathlessness severity	IRT (Rasch) Residual anova	uniform non-uniform	COPD: 123 Int lung disease: 129 CHF: 106 Total: 358	11 of 34 (32%)	no	no	non-invariant items deleted	dq
Yu, Yu & Ahn, 2007	SF-36 PF, MH	functioning; Mental health	MIMIC	uniform	Hypertension: 3156 Rheumatism:1654 Diabetes: 816 Res dis: 987 Dep: 864 Total: 8077	4 of 15 (27%)	yes	no	caution when comparing with hypertension; otherwise no adjustment	vs

Notes: n.n: not necessary; [1]conditions abbreviations: Neuro: neurological disorders; musco: musculoskeletal disorders; obst.: obstetrics; gyn: gynaecological disorder; Med comp: medically complex; MS: Multiple sclerosis; ALS: amyotrophic lateral sclerosis; TBI: traumatic brain injury; Cardio: cardiovascular disorder; med: medical; indust: industrial; CPM: chronic pain management; Rheuma: rheumatism; dep: depression; PD: Parkinson's disease; OA: Osteoarthritis; LSP: Late-onset sequels of poliomyelitis; RA: rheumatoid arthritis; PAD: peripheral arterial disease; CHF: chronic heart failure; Res dis: respiratory disease; [2]status of questionnaire: vq: validated questionnaire; vs: validiated scale; dq: questionnaire under development; ib: item bank;
