# Supplementary material for: Measurement invariance across chronic conditions: a systematic review and an empirical investigation of the Health Education Impact Questionnaire (heiQ™)
Source: Health Qual Life Outcomes. 2014 Apr 23;12:56. doi: 10.1186/1477-7525-12-56 (PMC4021509; doi:10.1186/1477-7525-12-56)
Supplement: Additional file 2: Table S2 — True mean differences (PIDIFF) and impact of non-invariant items on latent (ESSI-PI; white) and composite (ESPI-ALL, ESPI-RES, grey) mean differences. [file 1477-7525-12-56-S2.pdf]

Table A2

True mean differences (PI_{DIFF}) and Impact of non-invariant Items on latent ($ES_{SI\text{-}PI}$; white) and composite ($ES_{PI\text{-}ALL}$, $ES_{PI\text{-}RES}$, grey) mean differences

**Disease group**		**Ortho**	**Rheu**	**Asthma**	**COPD**	**Cancer**	
**Health-directed behavior**							
**Ortho**			<0.01	0.01	0.10	0.09	**$ES_{PI\text{-}ALL}$**
			[a]	[a]	<0.01	[a]	**$ES_{PI\text{-}RED}$**
**Rheu**	**PI_{Diff}**	-0.15		<0.01	0.10	0.10	**$ES_{PI\text{-}ALL}$**
	**$ES_{SI\text{-}PI}$**	>-0.01		[a]	0.011	[a]	**$ES_{PI\text{-}RED}$**
**Asthma**	**PI_{Diff}**	-0.01	0.139		0.08	0.10	**$ES_{PI\text{-}ALL}$**
	**$ES_{SI\text{-}PI}$**	>-0.01	0.000		0.01	[a]	**$ES_{PI\text{-}RED}$**
**COPD**	**PI_{Diff}**	0.20	0.36	0.18		0.19	**$ES_{PI\text{-}ALL}$**
	**$ES_{SI\text{-}PI}$**	0.11	0.12	0.10		0.15	**$ES_{PI\text{-}RED}$**
**Cancer**	**PI_{Diff}**	-0.68	-0.55	0.69	-0.89		
	**$ES_{SI\text{-}PI}$**	>-0.01	>-0.01	>-0.01	-0.12		
**Constructive attitudes and approaches**							
**Ortho**			0.03	0.02	0.01	0.02	**$ES_{PI\text{-}ALL}$**
			<0.01	[a]	0.03	[a]	**$ES_{PI\text{-}RED}$**
**Rheu**	**PI_{Diff}**	0.31		0.01	0.02	0.01	**$ES_{PI\text{-}ALL}$**
	**$ES_{SI\text{-}PI}$**	-0.05		0.01	0.02	0.08	**$ES_{PI\text{-}RED}$**
**Asthma**	**PI_{Diff}**	-0.24	-0.54		0.01	0.01	**$ES_{PI\text{-}ALL}$**
	**$ES_{SI\text{-}PI}$**	0.00	-0.05		0.01	[a]	**$ES_{PI\text{-}RED}$**
**COPD**	**PI_{Diff}**	0.02	-0.33	0.19		0.02	**$ES_{PI\text{-}ALL}$**
	**$ES_{SI\text{-}PI}$**	0.01	0.01	-0.03		0.05	**$ES_{PI\text{-}RED}$**
**Cancer**	**PI_{Diff}**	-0.32	-0.64	-0.07	-0.30		
	**$ES_{SI\text{-}PI}$**	0.00	0.05	0.00	0.04		
**Emotional distress**							
**Ortho**			0.04	0.01	<0.01	0.08	**$ES_{PI\text{-}ALL}$**
			0.03	0.02	0.01	0.011	**$ES_{PI\text{-}RED}$**
**Rheu**	**PI_{Diff}**	-0.69		0.06	0.03	0.03	**$ES_{PI\text{-}ALL}$**
	**$ES_{SI\text{-}PI}$**	0.01		[a]	[a]	0.02	**$ES_{PI\text{-}RED}$**
**Asthma**	**PI_{Diff}**	0.13	0.83		0.01	0.01	**$ES_{PI\text{-}ALL}$**
	**$ES_{SI\text{-}PI}$**	<0.01	<|-0.01|		[a]	0.01	**$ES_{PI\text{-}RED}$**
**COPD**	**PI_{Diff}**	-0.43	0.26	-0.49		0.07	**$ES_{PI\text{-}ALL}$**
	**$ES_{SI\text{-}PI}$**	0.01	0.000	<0.01		0.04	**$ES_{PI\text{-}RED}$**
**Cancer**	**PI_{Diff}**	0.01	0.80	-0.04	0.53		
	**$ES_{SI\text{-}PI}$**	-0.06	-0.06	-0.07	-0.06		
**Skill and technique acquisition**							

**Ortho**			<0.01	0.14	0.07	0.02	ES_{PI-ALL}
			0.02	0.06	0.03	0.02	ES_{PI-RED}
**Rheu**	PI_{Diff}	0.20		0.14	0.06	0.02	ES_{PI-ALL}
	ES_{SI-PI}	<|-0.01|		0.80	0.02	[a]	ES_{PI-RED}
**Asthma**	PI_{Diff}	-0.50	-0.69		0.05	0.09	ES_{PI-ALL}
	ES_{SI-PI}	-0.03	0.02		0.09	0.02	ES_{PI-RED}
**COPD**	PI_{Diff}	-0.20	-0.39	0.23		0.05	ES_{PI-ALL}
	ES_{SI-PI}	-0.02	-0.04	0.00		0.07	ES_{PI-RED}
**Cancer**	PI_{Diff}	-0.47	-0.67	0.00	-0.25		
	ES_{SI-PI}	<|-0.01|	0.00	0.00	0.02		

Health-Service Navigation

**Ortho**			0.00	0.14	0.16	0.14	ES_{PI-ALL}
			[a]	0.07	0.09	0.03	ES_{PI-RED}
**Rheu**	PI_{Diff}	0.25		0.14	0.15	0.14	ES_{PI-ALL}
	ES_{SI-PI}	<|-0.01|		0.02	0.04	0.02	ES_{PI-RED}
**Asthma**	PI_{Diff}	-0.06	-0.31		0.01	0.01	ES_{PI-ALL}
	ES_{SI-PI}	0.13	0.12		0.01	0.06	ES_{PI-RED}
**COPD**	PI_{Diff}	-0.07	-0.31	-0.01		0.03	ES_{PI-ALL}
	ES_{SI-PI}	0.13	0.12	-0.02		0.07	ES_{PI-RED}
**Cancer**	PI_{Diff}	-0.48	-0.72	-0.42	-0.41		
	ES_{SI-PI}	0.15	0.14	0.01	0.01		

Notes: Ortho: orthopedic condition; Rheu: rheumatism; PI_{Diff}: Estimations of latent mean differences in partial invariance models; ES_{SI-PI}: Difference between estimations of latent mean differences between strict and partial invariance models; ES_{PI-ALL}: Difference between latent mean differences in partial invariance models and composite mean differences using all items of a scale; ES_{PI-RED}: Difference between latent mean differences in partial invariance models and composite mean differences using only items with pairwise non-invariant parameters; [a] no item with DIF between groups
